# The Attack on the Matron of the London

**Published:** 1890-09-06

**Authors:** 


### The Attack on the Matron of the London.

### THE GOVERNORS SUPPORT THE HOUSE COMMITTEE.

At the quarterly meeting of governors at the London Hospital held on Wednesday, Mr. J. H. Buxton in the chair, Mrs. Hunter, who spoke well, opposed the adoption of the report of the House Committee containing the resolution of sympathy and confidence in the matron. Her chief grounds of objection were that the evidence given before the Lords' Committee was not generally available, and it would, therefore, be wrong to commit the governors to an approval of the matron's action, and that of the House Committee in supporting her absolutely. She stated that the night nurses' food consisted chiefly of pickled mackerel and sardines, with bread and butter *ad lib.* Holidays were too few; a nurse in training got only three weeks' holiday during her first two years. She denied that she was in any way connected with an organised opposition to the London Hospital.

Mr. Hole, who seconded Mrs. Hunter's amendment, objected to the transfer of any power from the House Governor. He said that the matron was absent each week three days out of seven without leaving any responsible head to govern in her absence. He desired the House Committee to be made more democratic by the addition of five tradesmen residing in the Whitechapel Road. These business men could manage the hospital properly.

In reply, Sir Edmund Currie said that the opposition to the matron was organised, and in proof of the unfounded character of the charges he read the evidence of Dr. Fenwick, Dr. S. Mackenzie, and Mr. Treves. He said all the opposition arose from a disease which attacked all hospitals alike, viz., the belief existing in some quarters that certain ladies were born nurses. There were 239 nurses engaged at the London, and out of them a proportion must fail from illness or incapacity. Sir Edmund Currie declared that the committee had faith in their matron, and that no one stands higher in the hospital world than Miss Lückes. It was false to state she was away three days out of seven.

Sir F. Nicholson declared he had never met a man or woman so devoted, conscientious, or efficient as their matron was. Miss Lückes worked fifteen hours a-day at the London. He had Miss Nightingale's authority to state for the information of the Governors and the public that she had satisfied herself that the charges made were without foundation, and that no lady stood higher in nursing circles in England than Miss Lückes does.

In reply to a question, the Chairman said that there were nine members of the House Committee present when the resolution of confidence and sympathy with the matron was passed, and it was carried unanimously. Every year the position of the nurses improved, and it would undoubtedly continue to improve. Up to five or six years ago the nurses were not properly fed, but since the opening of the Nursing Home in 1884 matters had greatly improved, till, at the present time, no nurses were so well fed and cared for on the whole as those at the London. The House Committee desired to extend the holidays from fourteen days to twenty-one days in each year. On a division, the amendment was rejected, and the report adopted with only three dissentients.

Mr. Burdett gave notice that at the next Court he should move: "That in regard to the recent and disparaging attacks upon the management of the London Hospital, this meeting of Governors sees no cause for witholding the confidence they have always reposed in the efficiency and integrity of the House Committee and its officers, with whom, while assuring them of their confidence, they desire to express their sympathy." He pointed out that in November the Governors would be in no better position to express an opinion than they were to-day, as the Lords' Committee would not have then reported, nor were they likely to report under two years. Whilst he accepted the disclaimer of Mrs. Hunter and her friends that they had no knowledge of a conspiracy, there was no doubt that an organized attempt had been made to discredit the matron of the London Hospital and the Nursing System there.

